# Mapping Europe into local climate zones

**DOI:** 10.1371/journal.pone.0214474

**Published:** 2019-04-24

**Authors:** Matthias Demuzere, Benjamin Bechtel, Ariane Middel, Gerald Mills

**Affiliations:** 1 Department of Environment, Laboratory of Hydrology and Water Management, Ghent University, Ghent, Belgium; 2 Department of Geography, Ruhr-University Bochum, Bochum, Germany; 3 Kode VOF, Ghent, Belgium; 4 Institute of Geography, University of Hamburg, Hamburg, Germany; 5 School of Arts, Media and Engineering, Arizona State University, Tempe, Arizona, United States of America; 6 School of Computing, Informatics, and Decision Systems Engineering, Arizona State University, Tempe, Arizona, United States of America; 7 Urban Climate Research Center, Arizona State University, Tempe, Arizona, United States of America; 8 School of Geography, University College Dublin, Dublin, Ireland; Cardiff University, UNITED KINGDOM

## Abstract

Cities are major drivers of environmental change at all scales and are especially at risk from the ensuing effects, which include poor air quality, flooding and heat waves. Typically, these issues are studied on a city-by-city basis owing to the spatial complexity of built landscapes, local topography and emission patterns. However, to ensure knowledge sharing and to integrate local-scale processes with regional and global scale modelling initiatives, there is a pressing need for a world-wide database on cities that is suited for environmental studies. In this paper we present a European database that has a particular focus on characterising urbanised landscapes. It has been derived using tools and techniques developed as part of the World Urban Database and Access Portal Tools (WUDAPT) project, which has the goal of acquiring and disseminating climate-relevant information on cities worldwide. The European map is the first major step toward creating a global database on cities that can be integrated with existing topographic and natural land-cover databases to support modelling initiatives.

## Introduction

It is a truism to state that cities occupy a very small proportion of the terrestrial landscape yet are both major drivers of climate change [1] and a focus for associated risks [2]. There are many examples to illustrate; for example, the European heat event of 2003 (which caused 70,000 premature deaths over a three month period), may have been driven by large scale processes caused in part by climate change [3, 4] but its impact was concentrated in cities where the urban heat island enhanced the hazard and housing practice exposed the vulnerable [5, 6]. Dealing with spatial hierarchies of cause and effect poses a real difficulty for global climate science. The fifth phase of the Climate Model Intercomparison Project (CMIP5) atmosphere-ocean global climate models or earth system models had a typical horizontal resolution of 150 km [7], 1-2 orders of magnitude larger than most cities. And even though the High Resolution Model Intercomparison Project (HighResMIP) for CMIP6 targets resolutions below 100 km [8], the consequence remains that cities are mostly ignored as physical entities in global climate models. With the exception of the ground-breaking research by [9] who performed a near-global climate simulation at a horizontal grid spacing of 930 m, most part of the impact of global climate change on cities is inferred from more spatially detailed projections using statistical or dynamical ‘downscaling’ techniques [10–14]. At the same time, [15] state that a ‘scientific evidence base in each urban center is essential for effective adaptation action’ (p540) while acknowledging the limits to ‘understanding and predicting impacts of climate change at a fine-grained geographic and sectoral scale’ (p550). An important component of this evidence base would be a climate-relevant database at a suitable scale that is derived using an internationally consistent methodology. This would go beyond the urban mask approach that simply categorises land-cover into urban and non-urban [16] and permits both the transfer of knowledge between cities and the application of climate models at urban scales.

**Fig 1:**
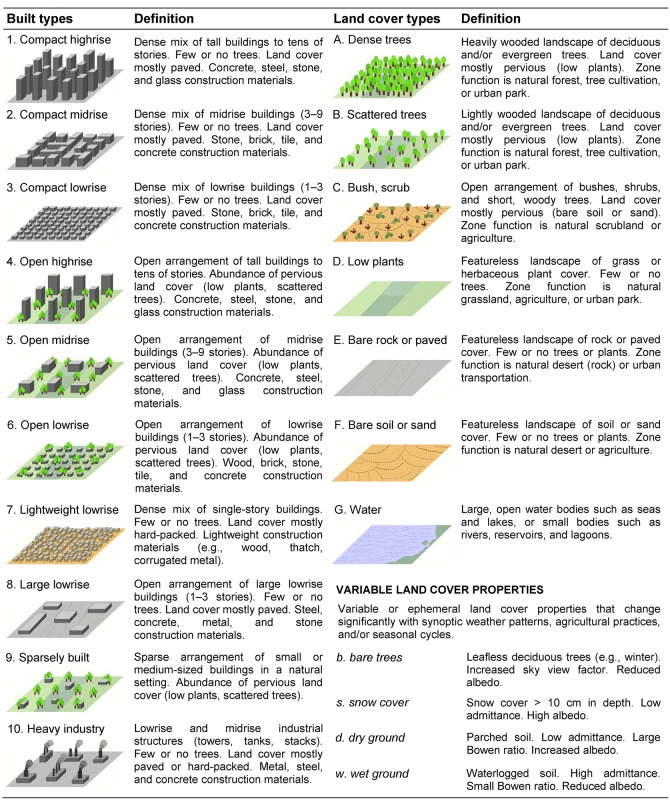
Abridged definitions for local climate zones [18]. ©American Meteorological Society. Used with permission.

The World Urban Database and Access Portal Tools (WUDAPT) project has as its goal the acquisition, storage and dissemination of data on cities worldwide and the development of tools to support climate research [17]. These data must account for aspects of physical form (i.e. urban land-cover, construction materials and the geometry of buildings) and functions (i.e. the transportation, energy usage, generation of waste products) that sustain human activities. However, it takes a pragmatic approach to the acquisition and organisation of data that recognises both the great variation in information currently available for different cities and the potential to generate consistent urban information using novel approaches. The Local Climate Zone (LCZ) typology [18] has been adopted as a baseline description of cities as it categorises distinct landscapes at a scale of about 1 km^2^ into recognisable types that can be linked to important surface parameters (known as urban canopy parameters, UCPs). Seventeen LCZ types exist, 10 of which can be described as ‘urban’ (Fig 1). Critically, each of these urban types is associated with numerical descriptions of key variables (termed urban canopy parameters, see Table 1) that can be used to model atmospheric responses to urbanisation [17, 18]. Moreover, because the LCZ scheme is a universal classification, the WUDAPT protocol is applicable to all cities thereby providing both a common platform for knowledge exchange and a pathway to model applications in cities where there is little data infrastructure. This protocol includes: selecting a spatial domain that contains a city of interest; creating training areas (TAs) that identify exemplars for each LCZ type present and; using available Landsat 8 images to classify the entire domain into LCZ types based on the training areas [19, 20]. To this point the WUDAPT process has been applied to individual cities and their surroundings; currently there are almost 100 cities worldwide and only few regions (e.g. Belgium [21]) available in the database. In addition, work has been done to test the idea of ‘transferability’, where information from one city is used to categorise a different independent city, thereby testing the universality of the LCZ framework as an urban typology [22]. However, these city-by-city approaches will not result in a database that could support urban decision-making globally in a reasonable time frame.

**Table 1:** Some of the parameter values associated with LCZ types from [18]. Columns represent the percentage of built (λ_*B*_ [%], ratio of building plan area to total plan area), impervious (λ_*I*_ [%], ratio of impervious plan area (paved, rock) to total plan area) and vegetated (λ_*V*_ [%], ratio of pervious plan area (bare soil, vegetation, water) to total plan area) land-cover and mean height of roughness elements (H [m], geometric average of building heights (LCZs 1–10) and tree/plant heights), sky view factor (SVF) and anthropogenic heat flux (AHF [W m^−2^]). The last column presents the total impervious surface density (IMD [%]), calculated as the sum of the outer ranges of λ_*B*_ and λ_*I*_.

LCZ	λ_*B*_	λ_*I*_	λ_*V*_	H	SVF	AHF	IMD
1. Compact high-rise	40–60	40–60	<10	>25	0.2–0.4	50–300	>80
2. Compact midrise	40–70	30–50	<20	10–25	0.3–0.6	<75	>70
3. Compact low-rise	40–70	20–50	<30	3–10	0.2–0.6	<75	>60
4. Open high-rise	20–40	30–40	30–40	>25	0.5–0.7	<50	50–80
5. Open midrise	20–40	30–50	20–40	10–25	0.5–0.8	<25	50–80
6. Open low-rise	20–40	20–50	30–60	3–10	0.6–0.9	<25	40-90
7. Lightweight low-rise	60–90	<20	<30	2–4	0.2–0.5	<35	>60
8. Large low-rise	30–50	40–50	<20	3–10	>0.7	<50	>70
9. Sparsely built	10–20	<20	60–80	3–10	>0.8	<10	10-40
10. Heavy industry	20–30	20–40	40–50	5–15	0.6–0.9	>300	>40
A. Dense trees	<10	<10	>90	3–30	<0.4	0	<20
B. Scattered trees	<10	<10	>90	3–15	0.5–0.8	0	<20
C. Bush, scrub	<10	<10	>90	<2	0.7–0.9	0	<20
D. Low plants	<10	<10	>90	<1	>0.9	0	<20
E. Bare rock or paved	<10	>90	<10	<0.25	>0.9	0	>90
F. Bare soil or sand	<10	<10	>90	<0.25	>0.9	0	<20
G. Water	<10	<10	>90	–	>0.9	0	<20

This paper outlines an approach to generate a continental scale LCZ map that goes beyond city boundaries, climate zones and ecoregions. The major contribution of the current research is to upscale the current city-by-city approach to continental scales, which permits the generation of UCPs for domains of choice. In this paper, we present the methodology used to generate a LCZ map for Europe and evaluate these data against independently derived urban data.

## Materials and methods

The current protocol for generating LCZ maps in WUDAPT relies on an ‘off-line’ workflow that integrates training areas (TAs) and Landsat 8 (L8) imagery within the SAGA software package over a limited spatial domain [20]. The process relies on well defined TAs that exemplify the LCZ types present in the domain; each TA is identified using Google Earth images aided by the visual and numerical information provided in [18]. The TA dataset is used to extract spectral data from L8 images which is then used in a random forest classifier [23] to categorise the entire domain into LCZ types.

Here, we present two strategies to expand LCZ coverage rapidly. The first recognises that much of the information contained in the TA data for one city is transferable to other cities for which no TA data is available [22]. The second employs Google’s Earth Engine (EE)—a cloud-based platform for planetary-scale analysis [24]—to use its computational power, access to a range of geospatial datasets (including Landsat) and a large number of predefined functions and methods (including a random forest classifier). These datasets include Sentinel 1, Sentinel 2 and the Defense Meteorological Program (DMSP) Operational Linescan System (OLS) night-time lights product that will provide additional information for discriminating LCZ types. Incorporating more data sources and sensors from EE’s cloud storage combined with a strategic use of TA data allows us to ‘up-scale’ the current WUDAPT approach to regions and continents, while maintaining quality and high spatial resolution.

Since the materials and method section contains an extensive number of abbreviations referring to datasets, their providers, variables or other sources of information, a comprehensive list of abbreviations and their corresponding explanation is available in S1 Table.

### Training areas

A critical element in the WUDAPT workflow for generating LCZ maps is the training area (TA) data generated by urban experts [17, 20]. Creating suitable TA data is a time-demanding procedure, both because of the intrinsic nature of the task (i.e. the extent and heterogeneity of urban areas) and the ability of the urban expert to identify and digitise TAs consistently. To evaluate the quality of the LCZ map that is generated from TA data, the WUDAPT method measures accuracy by sub-dividing the complete TA data into train and test sets, using the former to predict LCZ types and comparing these to the types present in the test set. The TA data is sub-divided 25 times by random sampling and a range of accuracy values are obtained [17, 21]. Improving the quality of a LCZ map is an iterative process directed by these accuracy measures, which are improved by modifying the TA data and repeating the process. Nevertheless, experience has shown that the knowledge of the urban expert is key. The human influence experiment (HUMINEX, [25]) found large discrepancies between LCZ maps generated by different urban experts but also that improvements of up to 20% in overall accuracy were achieved when a combined TA dataset was created from those generated by a number of experts.

One of the distinguishing features of the LCZ scheme is its universality, that is, it should be as applicable to both Lagos and Paris, for example. This means that, in principle, it should be possible to classify the urban landscape for one city using the TA data generated for another. A caveat to this statement is that the recognition of LCZ types in satellite imagery is based partly on the background landscape and its seasonal behaviour. [22] found that LCZ accuracy improved considerably when a) the source of the training data is from a city in the same ‘urban ecoregion’ [26] as the city of interest and b) if the training areas from several cities are combined.

**Fig 2:**
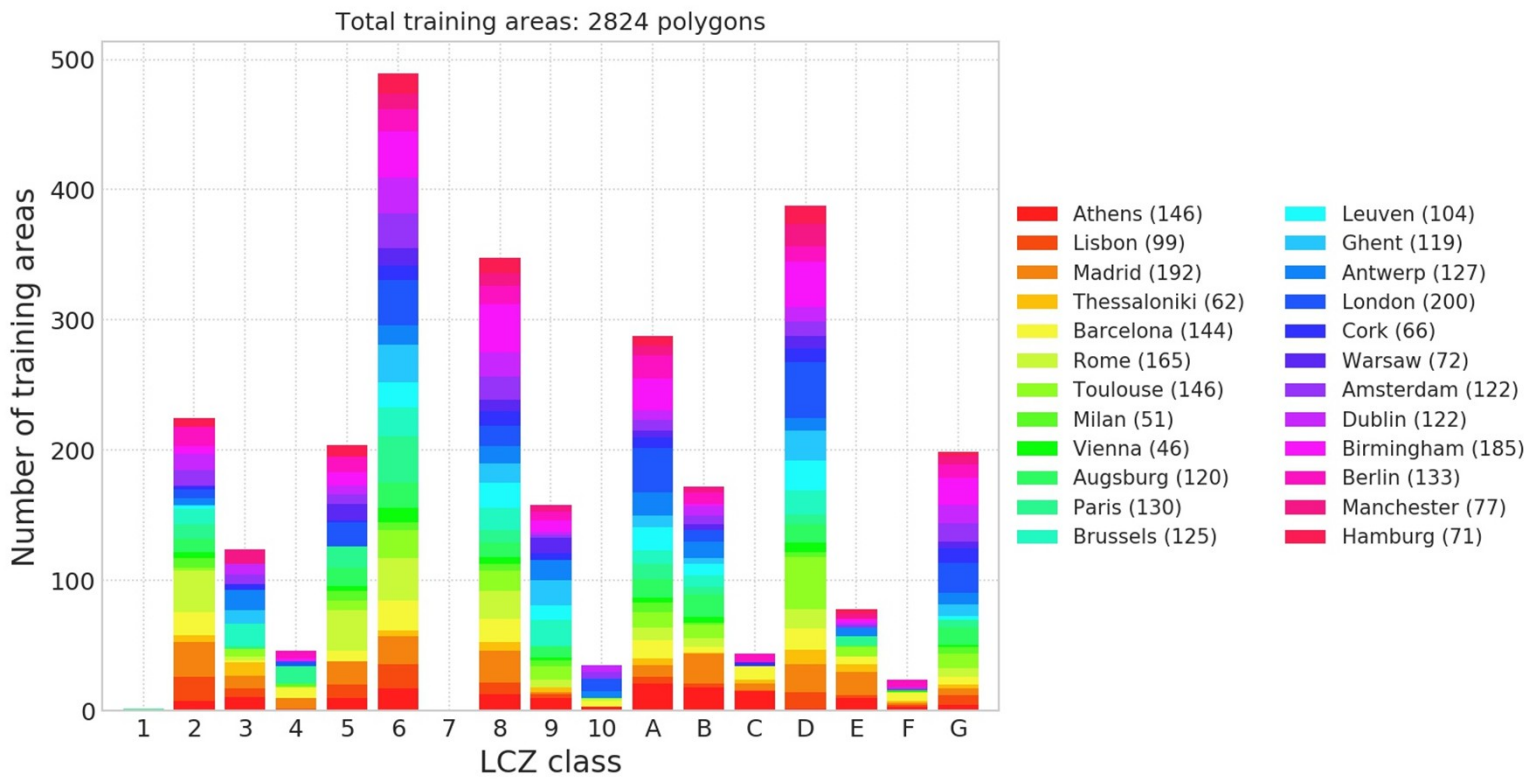
European training areas. Colours depict the number of training areas per LCZ class and city. Values between brackets are the total number of training areas per city.

**Fig 3:**
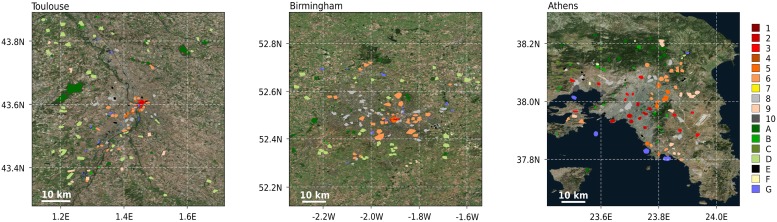
Illustration of training areas: Toulouse (France, left panel), Birmingham (United Kingdom, middle panel), and Athens (Greece, right panel). **Colours denote the LCZ classes**. Sources background map: Esri, DigitalGlobe, Earthstar Geographics, CNES/Airbus DS, GeoEye, USDA FSA, USGS, Aerogrid, IGN, IGP, and the GIS User Community.

Building further upon these findings, selected TAs from European cities available to the authors that passed WUDAPT’s automatic quality control [21] are combined into a large database (Table 2 and Figs 2 and 3). To ensure computational efficiency and more balanced training area sizes, TAs larger than 1 km^2^ (typically homogeneous water or forested areas) were detected and reduced in surface area to polygons with a radius of approximately 300 m [22].

**Table 2:** Available training areas for European cities.

City	Country	Published source or Author
Amsterdam	The Netherlands	Ran Wang
Antwerp	Belgium	[27]
Athens	Greece	[25]
Augsburg	Germany	[25, 28]
Barcelona	Spain	Joan Gilabert Mestre
Berlin	Germany	[25, 29]
Birmingham	United Kingdom	Ines Friedrich
Brussels	Belgium	[27]
Cork	Ireland	Paul Alexander
Dublin	Ireland	[20, 25, 30–32]
Ghent	Belgium	[27]
Hamburg	Germany	M. Kottas
Leuven	Belgium	[25]
Lisbon	Portugal	Max Anjos
London	United Kingdom	Niall Buckley
Madrid	Spain	[33, 34]
Manchester	United Kingdom	Micheal Foley
Milan	Italy	Maria Brovelli
Paris	France	[35]
Rome	Italy	Dragan Milosevic
Thessaloniki	Greece	Panagiotis Sismanidis
Toulouse	France	[35]
Vienna	Austria	[36]
Warsaw	Poland	Monika Tomaszewsk

### Earth observation data

The default WUDAPT workflow relies on Landsat 8 (L8) data as input to the random forest classifier [20]. In contrast, the research reported here benefits from EE’s client and server capacities [24] that offers a wealth of earth observation data in its public catalogue and a client library that can be used to compute derived products such as seasonal or multi-annual mean cloud-free composites or spectral indices. Building on the findings of [22], a total of 41 input features are used in the current study (Table 3). A summer (May to September, MtS), winter (November to March, NtM) and annual (January to December, JtD) composite is developed from L8 bands blue (B2), green (B3), red (B4), near infrared (B5), shortwave infrared (B6 and B7) and thermal infrared (B10 and B11). Also from L8, a large number of spectral indices are derived: the minimum and maximum Normalized Difference Vegetation Index (NDVI), the Biophysical Composition Index (BCI, [37]) using the Tasseled Cap transformation coefficients from [38], the mean Normalized Difference BAreness Index (NDBAI, [39]), the mean Enhanced Built-up and Bare land Index (EBBI, [40]), the mean Normalized Difference Water Index (NDWI, [41]) and the mean Normalized Difference Built Index (NDBI, [42]). The Normalized Difference Urban Index (NDUI) after [43] was introduced by combining the maximum of NDVI, NDWI and NDBI with the coarser resolution Defense Meteorological Satellite Program/Operational Linescan System (DMSP/OLS) nighttime light imagery [44]. Synthetic aperture radar (SAR) imagery is included via Sentinel-1, filtered by VV single co-polarisation, the Interferometric Wide swath acquisition mode and both ascending and descending orbits, and composited into a single image (hereafter referred to S1). From the S1 backscatter composite, an entropy and Geary’s C (a local measure of spatial association [45]) image is calculated, using a squared kernel of 5x5 pixels and a 9x9 spatial neighbourhood kernel respectively [22].

**Table 3:** Description of the input features. All products are selected for the period 2015-01-01 to 2017-12-31 (except indicated otherwise), and are resampled to a horizontal resolution of 100 m.

Input features	Source
Yearly (_JtD) and seasonal composites (_MtS, _NtM) (B2-7,B10,B11)	Landsat 8
Maximum and minimum NDVI	Landsat 8
BCI, NDBAI, EBBI, NDWI, NDBI	Landsat 8
NDUI^a^	Landsat 8 and NOAA DMSP-OLS Night-time lights
S1 backscatter and its entropy (_ent) and Geary’s C (_gc)	Sentinel-1
Global Forest Canopy Height (GFCH) ^b^	[46]
DTM, Slope and Aspect	USGS/GTOPO30
DSM	[47, 48]
DEM (DSM-DTM)	

^*a*^ The most recent night-time lights product is available for 2013.

^*b*^ This products reflects the state of 2005.

Finally, some auxiliary datasets are included such as the Global Forest Canopy Height (GFCH) product from [46], the GTOPO30 digital terrain model (DTM) and derived slope and aspect from the U.S. Geological Survey’s Earth Resources Observation and Science (EROS) Center, the ALOS World 3D global digital surface model (DSM) dataset [47, 48] and a digital elevation model (DEM) by subtracting the DTM from the DSM. Note that the full set of features are processed on a resolution of 100 m, following the default mapping resolution suggested by [20].

### Classification procedure and accuracy assessment

As a first step in the classification procedure, a feature importance ranking is performed using the permutation importance [49] from the randomForest v4.16-14 package available in R-project [50]. The input features for this procedure are obtained by sampling from the 2824 polygons that comprise the full TA dataset (Table 2, Fig 2); for each TA polygon, 10 pixels were randomly selected to ensure that the relative frequency of each LCZ class in the population was maintained. This resulted in 28,240 pixels (hereafter referred to as ‘All’ pixels) each containing information from 41 input features. As a first accuracy assessment, pixels are randomly split into train (70%) and test (30%) datasets which are classified into LCZ categories using the random forest classifier [23]. Initially this is done for all pixels and input features but this is at the limits of computational feasibility. Consequently, this exercise is repeated twice using: first, 33% of the 28,240 pixels (’Reduced’ pixels) with all 41 input features and; second, 100% of ‘All’ pixels containing the 10 most relevant input features.

The train and test split is repeated 25 times and for each realisation an accuracy is assessed according to the measures described in [22, 25, 51]: overall accuracy (OA), overall accuracy for the urban LCZ classes only (OA_*u*_), overall accuracy of the built versus natural LCZ classes only (OA_*bu*_), a weighted accuracy (OA_*w*_) and the class-wise metric F1. The overall accuracy denotes the percentage of correctly classified pixels. OA_*u*_ reflects the percentage of classified pixels from the urban LCZ classes only and OA_*bu*_ is the overall accuracy of the built versus natural LCZ classes only, ignoring their internal differentiation. The weighted accuracy (OA_*w*_) is a metric that is obtained by applying weights to the confusion matrix (available in Appendix A. of [25]) and accounts for the (dis)similarity between LCZ types. For example, LCZ 4 is most similar to the other open urban types (LCZs 5 and 6) leaving these pairs with higher weights compared to e.g. an urban and natural LCZ class pair. This results in penalising confusion between dissimilar types more than confusion between similar classes. Finally, the class-wise accuracy is evaluated using the F1 metric, which is a harmonic mean of the user’s and producer’s accuracy [51]. In addition, OA_*bu*_ is also used in combination with the Kappa coefficient and the True Positive Rate (TPR, positives correctly classified / total positives [52]) to verify whether the LCZ map is in line with a state of the art binary (urban / non-urban) global land cover product, described in the section below. Finally, EE’s random forest classifier is trained on all European TAs combined and used to categorise land-cover across the European continent into Local Climate Zone types.

### Evaluating large-scale LCZ maps

Each Local Climate Zone type is linked to a set of numerical values that describe a range of surface properties (Table 1) that include the average height buildings (H), impervious surface fraction (IMD), the anthropogenic heat flux (AHF) and sky view factor (SVF) for the ‘urban’ types (LCZ 1-10); note that Sparsely built (LCZ 9) is dominated by natural cover. These properties are especially relevant for studying urban areas as they are related to the generation of turbulence (H), the lack of water sources for evaporation (IMD), local heating of the atmosphere by human activities (AHF), and radiative trapping below roof level (SVF) (see [53]). These, along with others, are described as urban canopy parameters and are needed for capturing urban processes in atmospheric models. In order to evaluate the methodology presented here, we need equivalent independently obtained data to compare it with. In the following we present a variety of freely available published data sources that are used to evaluate the European LCZ map.

#### Urban land cover

The annual European Space Agency Climate Change Initiative (ESA CCI) land cover (LC) maps are available on a yearly basis from 1992 to 2015 at a spatial resolution of 300m [54]. These maps describe the Earth’s terrestrial surface in 37 original LC classes based on the United Nations Land Cover Classification System (UN-LCCS; [55]). In contrast to the natural classes, which are obtained using a wide range of satellite sensors (e.g. AVHRR, SPOT-VGT, MERIS FR/RR and Sentinel-3 OLCI/SLSTR), the single urban class is largely identified using two external datasets: the Global Human Settlement Layer (GHSL) [56] and the Global Urban Footprint (GUF) [54, 57]. GHSL is produced by the European Joint Research Center (JRC) based on an automated extraction of built-up areas from global Landsat imagery. In this dataset, the built-up area class abstraction is defined as the union of all the spatial units collected by the specific sensor and containing a roofed built structure or any portion of it [58, 59]. GUF is a recently released global binary settlement mask with a resolution of ∼12 m, and is derived from an automated Urban Footprint Processor used to analyse more than 180,000 TanDEM-X and TerraSAR-X radar images with 3 m ground resolution collected in 2011-2012 [57]. Note here that the SAR-based GUF approach might differ from the urban mask retrieved from optical data: the latter captures information from horizontal surfaces (e.g. parking lots and roads) but, as these features do not have a vertical component, they are not detected in GUF.

In order to compare the urban mask from ESA CCI with the European LCZ map, the latter is first re-projected and re-sampled to the 300 m ESA CCI resolution using a majority approach. Afterwards, both are converted into a binary product; for the LCZ map, all built LCZs (except Sparsely built (LCZ 9) which is predominantly natural) are converted to a single ‘urban’ class, and all remaining classes are considered as natural. The same approach is followed for ESA CCI. A quality assessment is then performed on a pixel-per-pixel basis for the entire European domain, and is described in terms of OA_*bu*_, the Kappa coefficient and the true positive rate.

#### Impervious surface cover (IMD) and building height (BH)

The Copernicus Land Monitoring Service (CLMS) as part of The European Environmental Agency (EEA) recently released an updated series of high resolution impervious density (IMD) datasets for the years 2006, 2009, 2012 and 2015 based on calibrated NDVI values. These maps provide an estimate of impermeable cover of soil (0-100%) at 20 m and 100 m resolutions [60]. CLMS have also released building height (BH) data for selected cities in the Urban Atlas project derived from IRS-P5 stereo images which is available at a spatial resolution of 10 m [61].

Both the IMD and BH datasets are used to extract urban canopy parameter information for the impervious surface fraction and height of the roughness elements [18], for each LCZ class from the European map. For the impervious surface fraction, the 100 m IMD product for 2015 is used, and ranges per LCZ are derived for the full coverage of the EEA IMD map. For the height of the roughness elements, the LCZ map is cropped to the corresponding cities’ extent of the BH data, and a spatial join is performed to extract the mean building height per built LCZ class, which is approximately comparable to H in Table 1.

#### Anthropogenic heat flux (AHF)

The AHF associated with each LCZ type [18] can be used to derive a dataset comparable to the annual AHF dataset provided by [62], which is available globally at a spatial resolution of 30 arc-seconds and temporal resolution of 1 hour. This product includes four heating components: energy loss, heating from the commercial, residential, and transportation sectors, heating from the industrial and agricultural sectors, and heating from human metabolism. For the current study, the annual average AHF is used for the European LCZ domain, and intersected with the 100 m LCZ map after a bilinear resampling to match resolutions. This is an approximate match with AHF in Table 1.

#### Sky view factor (SVF)

The SVF is a point-based measurement of the proportion of the sky vault that is observable from ground level. Here, the SVF (and other geometrical properties) are obtained for selected European cities using Google Street View (GSV) images that are examined using a big data and deep learning approach. First, a complete sample of GSV locations in each city is retrieved through the Google Maps API. For all locations, an image cube is downloaded in the form of six 90-degree field-of-view images that face upwards, downwards, north, east, south, and west [63]. The images are segmented by a convolutional neural network that was fine-tuned with GSV 90-degree images from cities around the world to yield six classes: sky, trees, buildings, impervious and pervious surfaces, and non-permanent objects [64]. Subsequently, each segmented image cube is projected onto a sphere, and fractional percentages of each class coverage on the sphere are calculated, representing the land cover composition as experienced by a pedestrian in a street canyon. In addition, the segmented upper half of the cube is projected into a hemispherical fish-eye to calculate the SVF using sky and non-sky pixels [65]. It is important to bear in mind that the GSV samples from the road network so that places that are not seen (e.g. parks and gardens) are not included.

To relate the point-based spherical fractions and SVFs to the LCZ maps, the datasets are spatially joined in ArcGIS, which yields average parameter values for all LCZ polygons that have processed GSV data [66]. Finally, the spherical fractions and SVFs are summarised by LCZ class for each city to calculate city-specific parameter means. These values are compared with SVF values from Table 1.

## Results

### Feature importance and accuracy assessment

**Fig 4:**
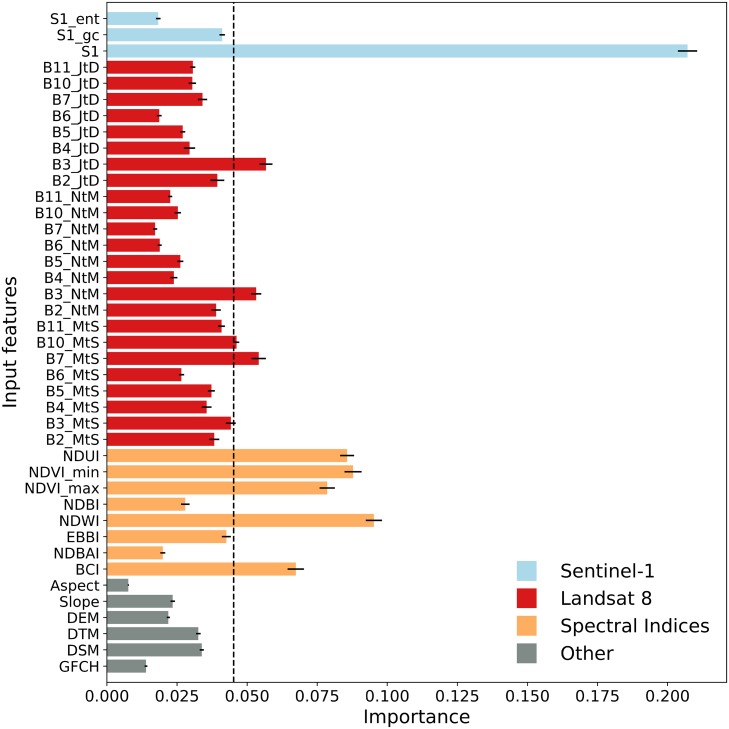
Input feature importance ranking. The vertical dashed line separates the top-10 features. The error bars are derived from the bootstrapping procedure. Note that the spectral indices are also derived from Landsat 8; they are given a different color for clarity.

The input feature importance ranking based on All pixels (28, 240) is presented in Fig 4. Sentinel-1 backscatter (S1) is by far the most important input feature, followed by those based on spectral ratios such as NDUI, minimum and maximum NDVI, NDWI and BCI. The top-10 list is completed by the annual (JtD) and winter (NtM) mean composite of L8’s green band (B3) and the summer mean composites of the shortwave infrared band (B7) and thermal infrared (B10). This result is very much in line with the work of [22], which tested the transferability of input features and TA data for 15 cities from across the world; this supports the view that the urban TA data provides a great deal of information that is generic to all urban landscapes.

**Fig 5:**
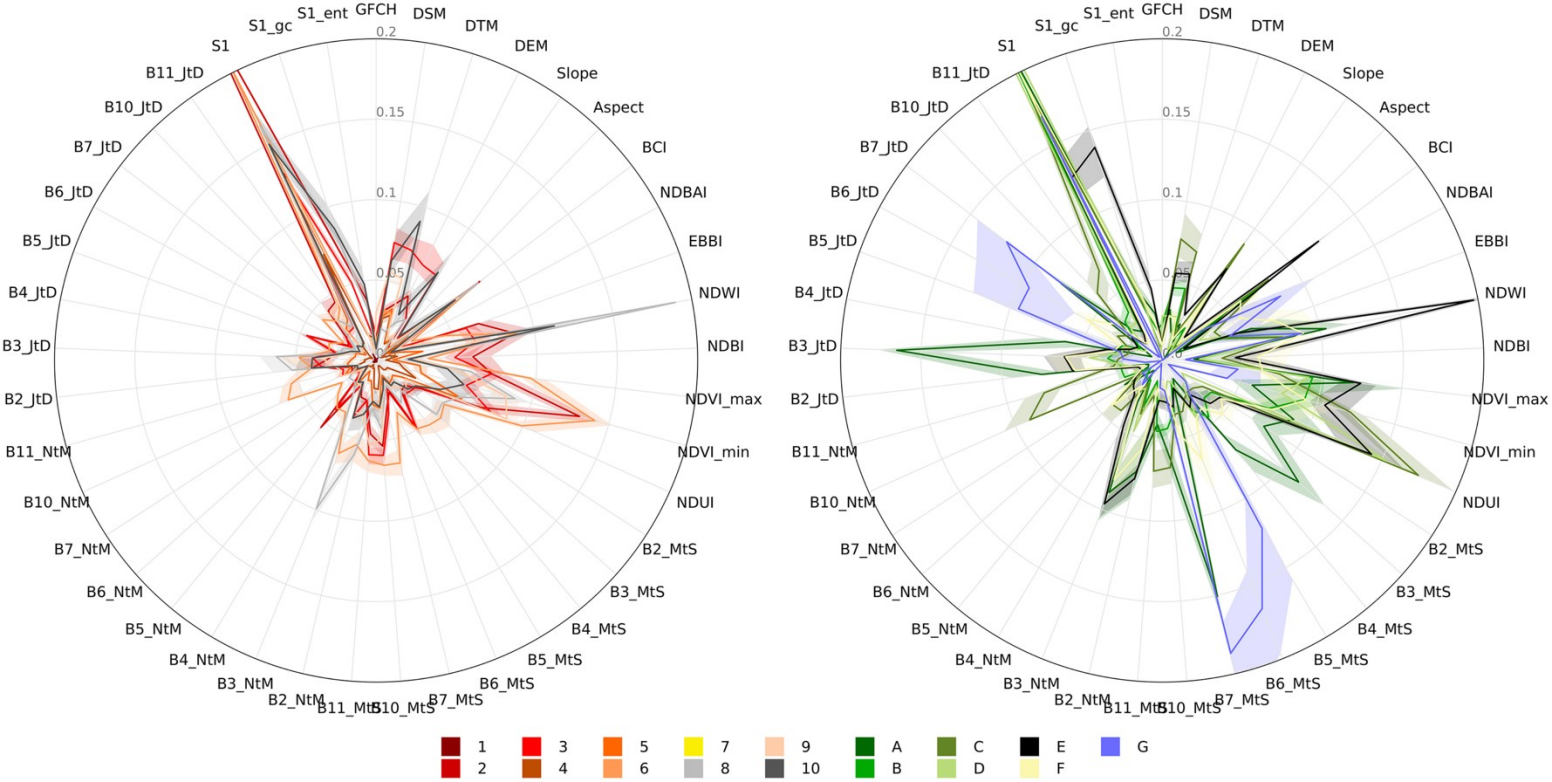
Feature importance ranking per LCZ class. Left panel: Built LCZs, right panel: Natural LCZs. Thick lines and shadings respectively present the importance mean and standard deviation from the bootstrapping.

While Fig 4 presents the relative importance of each input feature for all available LCZ types together, Fig 5 provides more in-depth information on the relative importance of input features for each LCZ type. As expected, S1 is the most important feature for most of the built-up and natural types. For the built LCZ classes, NDWI, minimum and maximum NDVI and NDUI are also relatively strong, although their signal varies depending on the building compactness and mean height that characterises each class. This class-wise analysis shows both the features that are useful for distinguishing among LCZ types and, conversely, LCZ types that are not well captured by the current input feature space. For example, the Compact high-rise class (LCZ 1), has extremely low importance rankings for all of the input features although this may partly be explained by the very low number of TAs for this class (Fig 3) and their small spatial extent; a similar observation can be made for the Open high-rise (LCZ 4) types. The natural LCZ types show more variability across input features, for example: Water (LCZ G) is best detected using L8’s infrared and shortwave infrared bands (B5, B6 and B7), Dense tree cover (LCZ A) using the green band of L8 (B3) and Bush/scrub cover (LCZ C) using L8’s thermal infrared bands (B10, B11), NDUI and minimum NDVI. Finally, Bare rock and paved area (LCZ E) has strong peaks in BCI, NDWI, NDUI, minimum and maximum NDVI and L8’s green band (B3).

**Fig 6:**
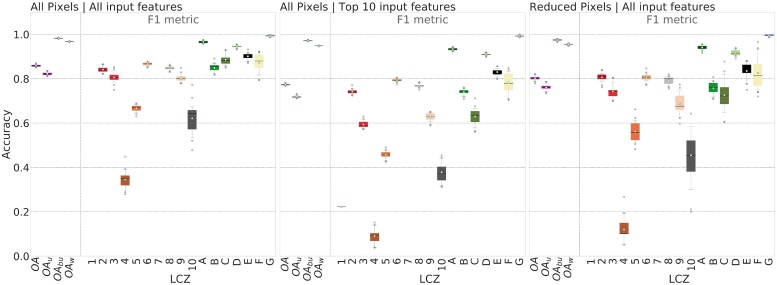
Classification accuracy assessment. Results are based on All pixels and all input features (left panel), All pixels and the top 10 input features (middle panel), and the reduced pixels and all input features (right panel).

Three experiments are performed to assess the accuracy of the LCZ predictions using all or part of the TA dataset and the input features; accuracy scores for each are depicted in Fig 6. In each experiment, 70% of the pixels are used to train and 30% to test; these pixels are selected by stratified (LCZ type) random sampling and this exercise is repeated 25 times allowing us to provide confidence intervals around the accuracy metrics.

Fig 6 (left) includes All pixels (28,240) and All input features (41) which generates scores >80% for all OA metrics. A similar observation can be made for the class-wise F1 metric, with the exception for LCZs 4 (Open high-rise), 5 (Open mid-rise) and 10 (Heavy industry). LCZ 1 (Compact high-rise) does not show as the few test pixels (from Paris and Brussels) are miss-classified as LCZ 2 (Compact mid-rise). Generally, the LCZ types 1 and 2, 4 and 5 are characterised by similar surface cover fractions and differ mainly by the height of their roughness features (>25 m and 3-10 m respectively, see Table 1). This points to a current limitation of the input feature space: apart from the S1 backscatter information, which responds to vertical elements on the landscape [67] and the DEM information (subject to errors [47, 48]), there is no input feature in the current suite that detects height variations. This is in line with the results of the 2017 IEEE GRSS Data Fusion Contest [34, 68], that found that the Open high-rise (LCZ 4) and Open mid-rise (LCZ 5) types were difficult to distinguish. To overcome this limitation, some of the teams in the Data Fusion Contest enriched their remotely sensed input features with building heights from OpenStreetMap (OSM) resulting in higher accuracy scores. However, [69] reported that including height information for Belgian cities did not significantly improve the LCZ mapping accuracy, mainly due to a mismatch in spatial resolution between the input feature sets. While there are limited domain height datasets available (e.g. Urban Atlas building height [61]), full coverage over the area of interest is needed to be included as an input feature.

Fig 6 also shows accuracy scores using the reduced pixel sample with all features (middle) and All pixels with the top 10 input features based on the importance ranking analysis (right) (Fig 4). Using fewer input features reduces the OA and OA_*u*_ values to below 80%. As before, lowest scores are obtained for LCZs 1, 4, 5 and 10 but there is also more confusion for LCZ 3 (Compact low-rise), LCZ 9 (Sparsely built) and LCZ C (Bush/scrub). On the other hand, using a smaller set of pixels in the TA dataset with all 41 input features generated OA scores close to or above 80%, and most F1 values are >75% except for LCZs 4, 5 and 10. From a computational perspective, these experiments indicate that it is better to sample less and use all input features than to sample more and use fewer inputs; this is the path taken here to create a European LCZ map.

### Local climate zone map

**Fig 7:**
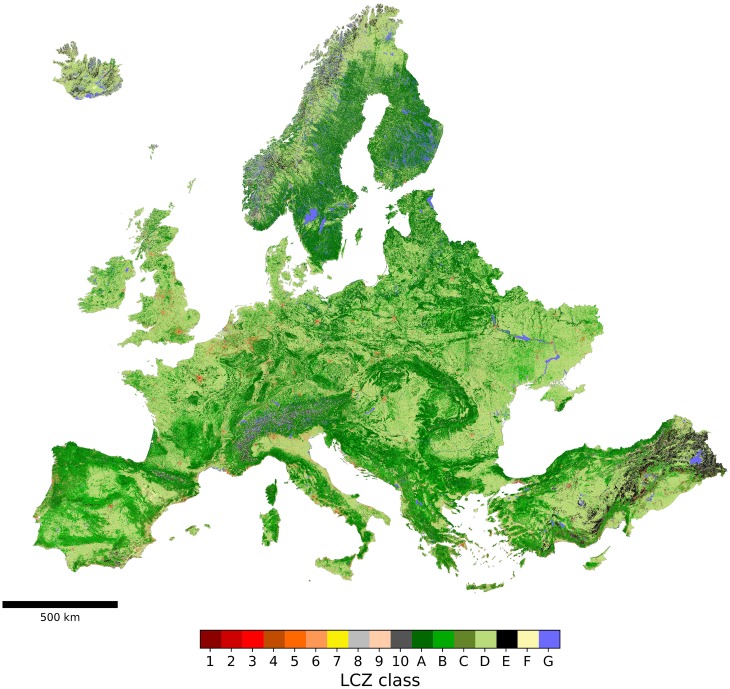
European local climate zone map. A selection of cities are depicted in more details in the Figures below.

The result of the European LCZ classification, based on the training of the random forest classifier in Earth Engine using the reduced set of pixels with all 41 input features is shown in Fig 7. The map displayed here is the raw outcome of the classifier, on a spatial resolution of 100 m, provided on the European ETRS89 / ETRS-LAEA (EPSG:3035) projection.

**Fig 8:**
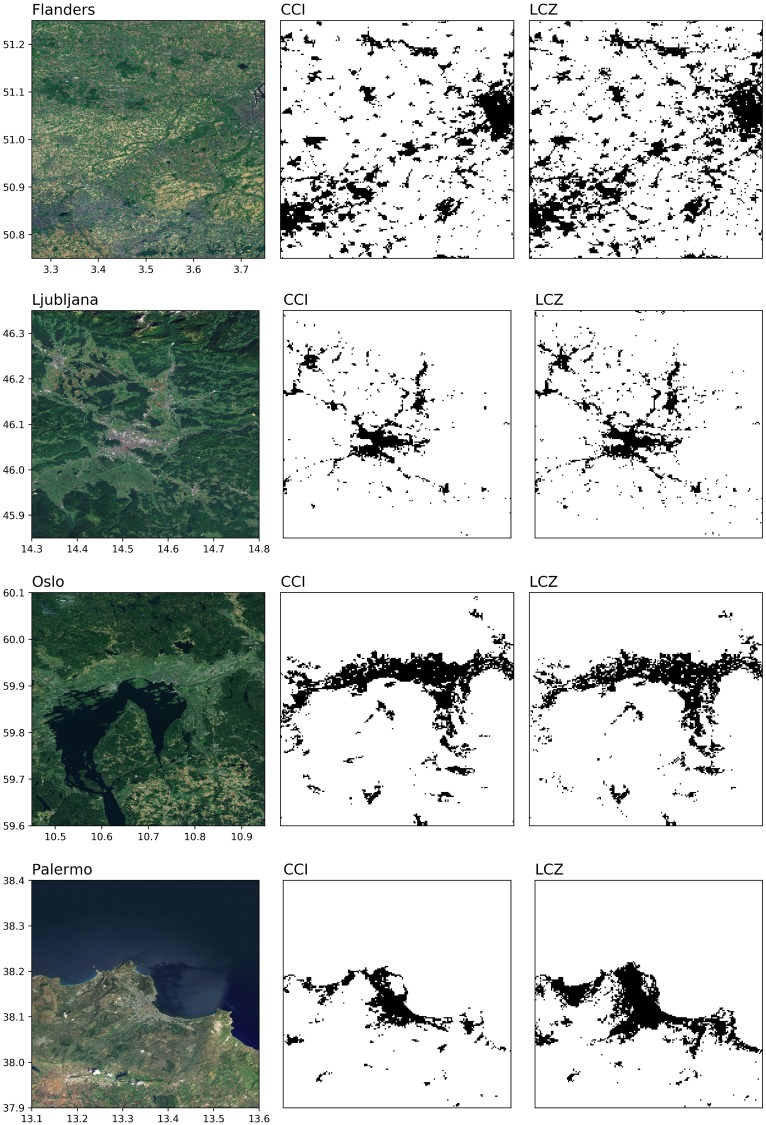
Binary urban maps extracted from ESA CCI and LCZ. Black refers to urban, white to non-urban. For the LCZs, classes 1-10 with the exception of 9 (Sparsely built) are considered urban. Sources background maps: Esri, DigitalGlobe, Earthstar Geographics, CNES/Airbus DS, GeoEye, USDA FSA, USGS, Aerogrid, IGN, IGP, and the GIS User Community.

As a preliminary evaluation, a binary urban/non-urban land cover comparison is made with the ESA CCI 2015 land cover product, taking this product as the ‘true’ reference. Fig 8 presents a first visual interpretation for four selected areas: (part of) Flanders (Belgium), Ljubljana (Slovenia), Oslo (Norway) and Palermo (Italy). One can see that in general, the urban extent is very similar between the two products and matches well with the urban areas as seen on the satellite imagery. If anything, the LCZ maps seem to produce more extensive urban areas, which is most apparent in the scattered urban pixels of the LCZ map for (part of) Flanders, as well as in the larger urban extent east and south of the city centre of Palermo (Fig 8, top and bottom row respectively). These are generally correspond to low-density low-rise built up areas that are not captured by ESA CCI but that are detected by the LCZ scheme.

**Fig 9:**
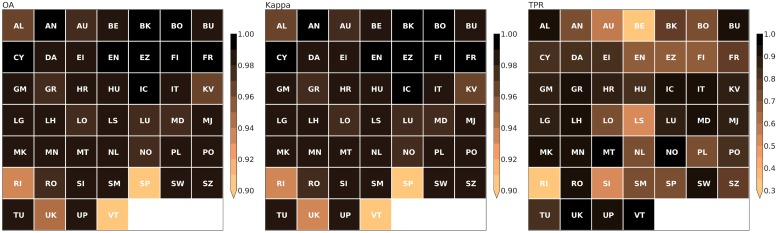
Accuracy assessment urban land cover for all European countries. Abbreviations refer to Large Scale International Boundary (LSIB) country codes: AL: Albania, AN: Andorra, AU: Austria, BE: Belgium, BK: Bosnia & Herzegovina, BO: Belarus, BU: Bulgaria, CY: Cyprus, DA: Denmark, EI: Ireland, EN: Estonia, EZ: Czech Republic, FI: Finland, FR: France, GM: Germany, GR: Greece, HR: Croatia, HU: Hungary, IC: Iceland, IT: Italy, KV: Kosovo, LG: Latvia, LH: Lithuania, LO: Slovakia, LS: Liechtenstein, LU: Luxembourg, MD: Moldova, MJ: Montenegro, MK: Macedonia, MN: Monaco, MT: Malta, NL: The Netherlands, NO: Norway, PL: Poland, PO: Portugal, RI: Serbia, RO: Romania, SI: Slovenia, SM: San Marino, SP: Spain, SW: Sweden, SZ: Switzerland, TU: Turkey, UK: United Kingdom, UP: Ukraine, VT: Vatican City.

The spatial accuracy of the European map was examined by calculating three performance measures for the LCZ cover in each European country, extracted using the Large Scale International Boundary (LSIB) country codes available in EE (Fig 9). For OA and Kappa, most accuracy scores are higher than 95%, except for Serbia (RI), Spain (SP), United Kingdom (UK) and Vatican City (VT). Yet given that there are substantially more natural than urban pixels across the domain, the true positive rate (TPR) is a much more stringent accuracy assessment criterion as it emphasises the correctly classified urban classes only. Here, TPR values tend to be lower but overall >70% accuracy was obtained. Interestingly, scores are lowest for Serbia and Belgium (BE) even though for the latter, four Belgian cities were included into the training process. After visual inspection, it is clear that ESA CCI is more conservative in its urban extent compared to the LCZ result. For the particular case of Belgian cities, ESA CCI underestimates some of the scattered urbanisation pattern that has been described elsewhere [70] (not shown).

### Urban canopy parameters

In the following, the derived Local Climate Zone map is spatially intersected with independently derived urban datasets that contain comparable canopy parameter values to those associated with the LCZ types (Table 1). This will allow us to a) evaluate the LCZ map and b) potentially fine-tune the existing generic ranges provided in Table 1, referred to as LCZ values in the following text.

#### Building height

**Fig 10:**
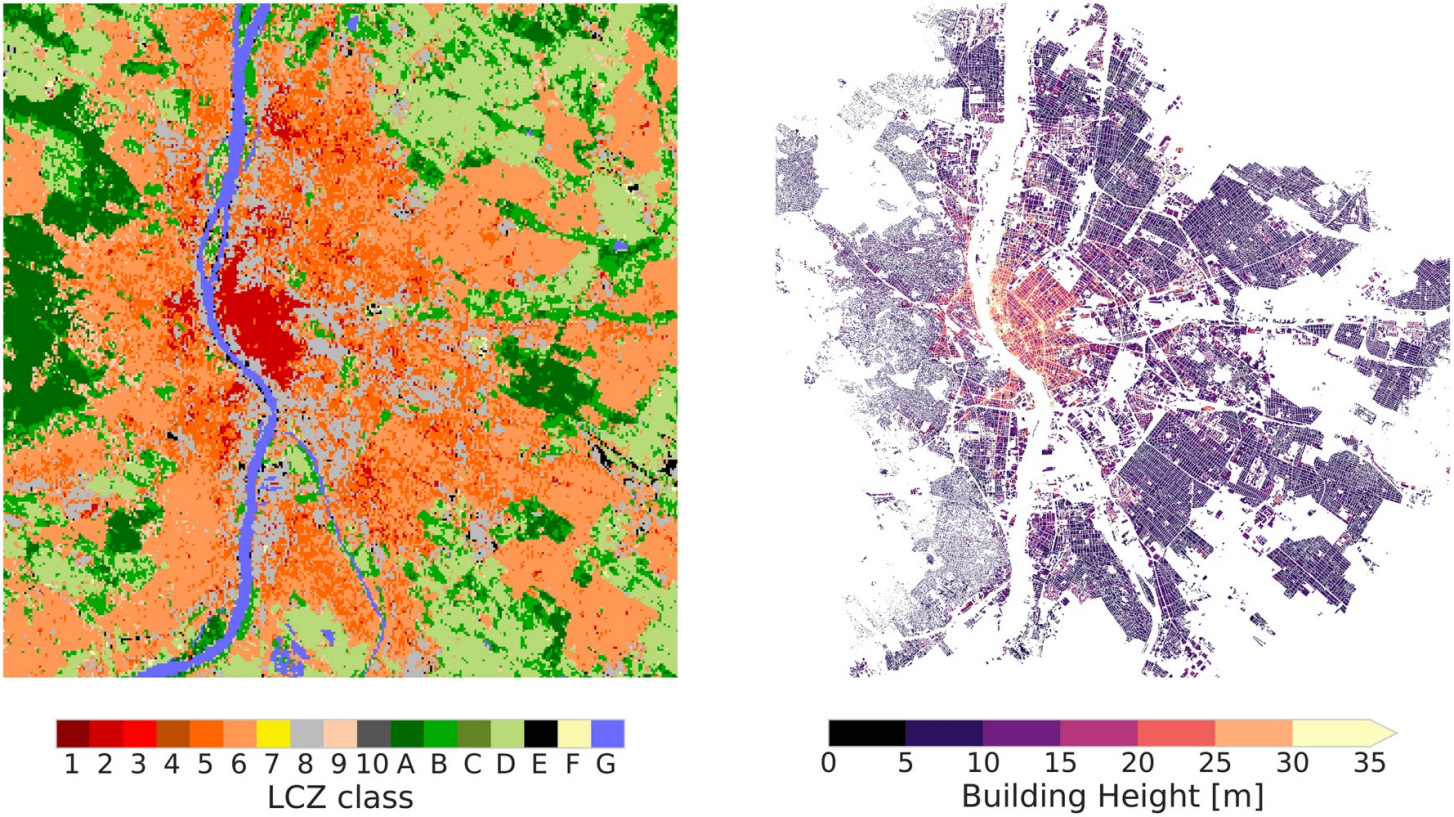
LCZ map (left panel) and EU Urban Atlas building height (right panel) for Budapest (Hungary).

Fig 10 depicts the LCZ map for the city of Budapest (Hungary), together with the Urban Atlas building height information. The city centre is characterised by Compact mid-rise cover (LCZ 2) with corresponding higher buildings (average of 16.9 m, see Table 4). This area is mainly surrounded by Open mid-rise (LCZ 5) and then Open low-rise (LCZ 6) with intervening areas of extensive Large low-rise (LCZ 8) associated with warehouses and/or shopping malls. The average derived heights for these zones are respectively 10, 7.6 and 11.6 m. For the case of Budapest, these values generally fall within the height of the LCZ roughness element ranges (Table 4).

**Table 4:** Mean building height (m) per built LCZ class for the Urban Atlas cities. Reference values from [18] are provided in the top row, mean and standard deviation (St. Dev.) across all Urban Atlas cities are provided on top of the individual city listings.

	LCZ 1	LCZ 2	LCZ 3	LCZ 4	LCZ 5	LCZ 6	LCZ 7	LCZ 8	LCZ 9	LCZ 10
[18]	>25	10-25	3-10	>25	10-25	3-10	2-4	3-10	3-10	5-15
**Mean**	25.0	14.7	11.2	11.8	10.6	7.9	-	10.5	7.3	12.0
**St. Dev**.	0.5	2.5	2.2	3.5	1.9	1.3	-	1.5	1.1	4.5
Amsterdam	-	14.1	11.1	12.3	12.1	8.2	-	10.1	8.6	10.6
Athens	-	14.4	9.6	18.6	11.4	8.5	-	12.4	7.4	23.3
Berlin	-	18.7	15.7	11.8	12.8	8.0	-	11.6	8.0	10.1
Bratislava	-	15.4	13.6	12.6	11.3	7.7	-	10.5	5.7	6.9
Brussels	24.4	15.2	11.8	12.2	11.5	9.0	-	11.2	8.9	-
Bucharest	-	11.0	7.7	11.6	9.8	6.4	-	9.0	5.9	-
Budapest	-	16.9	12.8	9.7	10.0	7.6	-	11.6	7.3	13.5
Copenhagen	-	12.9	14.3	7.3	7.8	6.6	-	8.1	6.5	15.4
Dublin	-	12.2	8.7	9.7	8.8	6.4	-	8.8	6.4	10.1
Helsinki	-	17.0	12.6	8.5	8.3	6.6	-	10.2	6.2	16.9
Lefkosia	-	9.0	7.5	-	8.0	7.3	-	8.1	7.0	5.8
Lisbon	-	13.5	9.0	21.0	12.3	7.6	-	11.8	6.7	10.4
Ljubljana	-	14.1	11.0	7.4	8.0	6.1	-	9.6	5.2	-
London	-	15.4	7.7	13.0	9.5	7.1	-	9.6	7.1	12.2
Luxembourg City	-	14.8	10.4	8.2	10.5	8.8	-	11.5	9.3	-
Madrid	-	16.8	11.6	18.6	15.0	10.6	-	12.5	9.2	10.7
Oslo	-	14.8	12.0	7.1	7.8	6.8	-	9.2	6.6	21.9
Paris	25.6	17.7	11.5	14.8	12.2	8.1	-	10.8	8.2	14.5
Prague	-	18.7	15.1	12.8	12.5	9.3	-	11.6	8.0	-
Reykjavik	-	8.2	6.8	6.2	6.8	5.5	-	6.2	5.2	8.7
Riga	-	16.5	10.8	11.6	11.3	10.6	-	10.7	8.8	8.0
Rome	-	17.1	11.1	13.9	13.7	9.3	-	13.5	9.0	14.5
Sofia	-	14.6	12.7	13.7	13.1	10.2	-	12.2	8.5	5.5
Stockholm	-	16.1	12.0	8.7	9.6	6.7	-	10.2	6.4	11.8
Tallinn	-	14.6	12.2	13.7	11.5	8.3	-	9.5	6.6	9.3
Vienna	-	16.7	11.7	12.8	10.4	7.1	-	10.8	6.9	-
Vilnius	-	12.0	11.6	12.2	11.3	8.7	-	10.9	6.7	-
Warsaw	-	16.0	12.2	10.7	11.0	8.2	-	11.5	7.9	-
Zagreb	-	12.8	8.7	9.1	10.4	7.6	-	10.6	6.8	-

Table 4 shows the results for all other Urban Atlas cities that provide information on building height. The few Compact high-rise (LCZ 1) pixels in Brussels and Paris have a mean building height of 25 m, which is in line with the lower limit of LCZ values, shown in the top row. Overall, most other buildings heights are also in line with the LCZ values, except for Compact low-rise (LCZ 3) (slightly too high) and especially Open high-rise (LCZ 4), which has too low building heights (11.8 ± 3.5 m) compared to the LCZ value >25 m. This is in line with the results of Fig 6 that revealed the lowest accuracy values for the LCZ 4 type, because of insufficient underlying input information on building height, and hence a general tendency to confuse this class with Open mid-rise (LCZ 5). A similar observation can be made for Compact low-rise (LCZ 3) and its nearest type (Compact mid-rise, LCZ 2) with taller buildings.

#### Impervious surface fraction (IMD) and anthropogenic heat flux (AHF)

**Fig 11:**
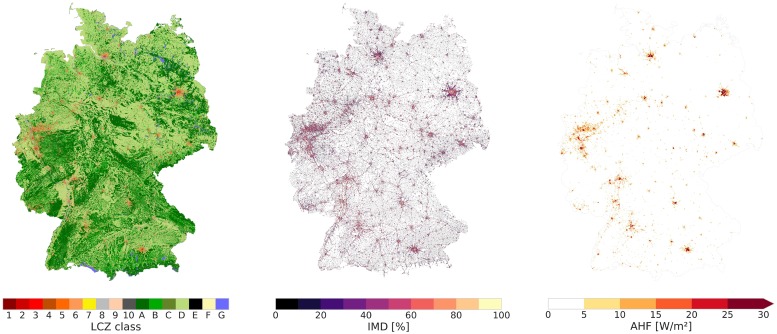
LCZ map, impervious density (IMD, %) and mean annual anthropogenic heat flux (AHF, *W*/*m*^2^) for Germany.

Here the LCZ map and its associated properties are compared with impervious density information (IMD, [60]) and the annual mean anthropogenic heat flux (AHF, [62]). To illustrate the spatial correspondence Fig 11 shows the LCZ map for Germany alongside IMD and AHF; one can see that the compact LCZ types (1 to 3 and 8) have high IMD values (see for example, the city centres of Hamburg, Berlin and Munich) and that these are also areas of high AHF (± 30 W m^−2^).

The statistics for IMD and AHF across the European domain are tabulated per LCZ class in Table 5. The most compact LCZ types are 1, 2, 3, 8 and 10, with impervious fractions well above 50% which is generally in line with the LCZ values. In contrast, the fractions for the other built classes are typically lower, except for the Sparsely built type (LCZ 9). For the natural classes, some classes seem to suffer some confusion; for example, the impervious surface fraction estimated for Bare soil/sand (LCZ F) is 33.14 ± 11.7% and that for Water (LCZ G) is 13.74 ± 17.45%. These results could be a byproduct of spatial resolution, for example small or narrow water bodies may not captured by the horizontal resolution of the 100 m LCZ map [51].

**Table 5:** Impervious density (IMD) and mean annual anthropogenic heat flux (AHF) urban canopy parameters. Mean and standard deviation are calculated over the entire European domain (LCZ map) and are stratified per LCZ class. Reference values from [18] are provided as well.

	IMD		AHF	
	[18]	LCZ map	[18]	LCZ map
LCZ 1	>80	90.44	50–300	29.78
LCZ 2	>70	71.36 ± 8.52	<75	11.24 ± 9.27
LCZ 3	>60	62.17 ± 10.74	<75	5.21 ± 4.88
LCZ 4	50–80	39.87 ± 3.08	<50	5.81 ± 2.02
LCZ 5	50–80	45.09 ± 11.45	<25	6.44 ± 8.48
LCZ 6	40-90	26.83 ± 11.55	<25	2.39 ± 3.16
LCZ 7	>60	-	<35	-
LCZ 8	>70	58.53 ± 14.22	<50	3.49 ± 5.39
LCZ 9	10-40	12.85 ± 8.03	<10	0.73 ± 0.78
LCZ 10	>40	64.40 ± 11.76	>300	1.58 ± 0.68
LCZ A	<20	5.71 ± 8.1	0	0.37 ± 0.33
LCZ B	<20	7.60 ± 7.79	0	0.53 ± 0.67
LCZ C	<20	15.83 ± 6.51	0	0.49 ± 0.24
LCZ D	<20	8.76 ± 7.96	0	0.48 ± 0.44
LCZ E	>90	40.93 ± 19.87	0	0.73 ± 0.54
LCZ F	<20	33.14 ± 11.7	0	0.60 ± 0.4
LCZ G	<20	13.74 ± 17.45	0	0.47 ± 0.79

In [18] AHF represents fuel combustion and human activity and the range provided for each LCZ type (with the exception of Compact high-rise, LCZ 1) is only bounded by an upper threshold value (Table 1). This makes a direct comparison with [62] difficult, hence the values in Table 5 might serve as a first-order approximation for the annual heat flux density. Note that these values are expressed as an annual mean flux, even though its value varies significantly with season and time of the day. To tackle this issue, one could either extract the full hourly dataset from [62] or follow the methodology as proposed by [71] and applied by e.g. [13, 72].

#### Sky view factor (SVF)

**Fig 12:**
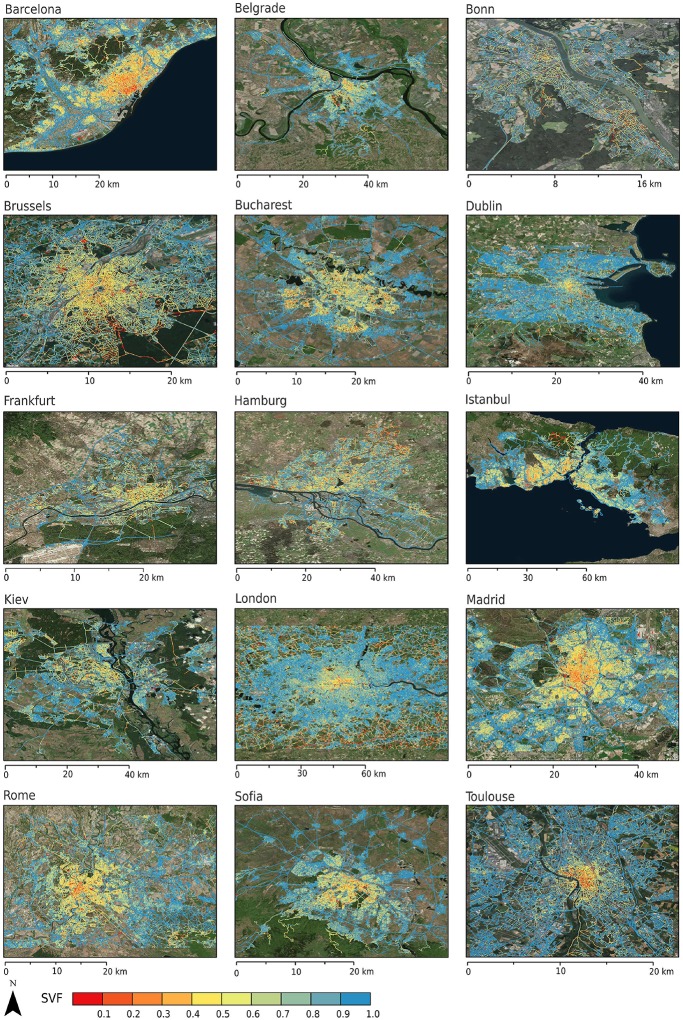
Sky view factors for 15 European cities. Sources background map: Esri, DigitalGlobe, Earthstar Geographics, CNES/Airbus DS, GeoEye, USDA FSA, USGS, Aerogrid, IGN, IGP, and the GIS User Community.

**Fig 13:**
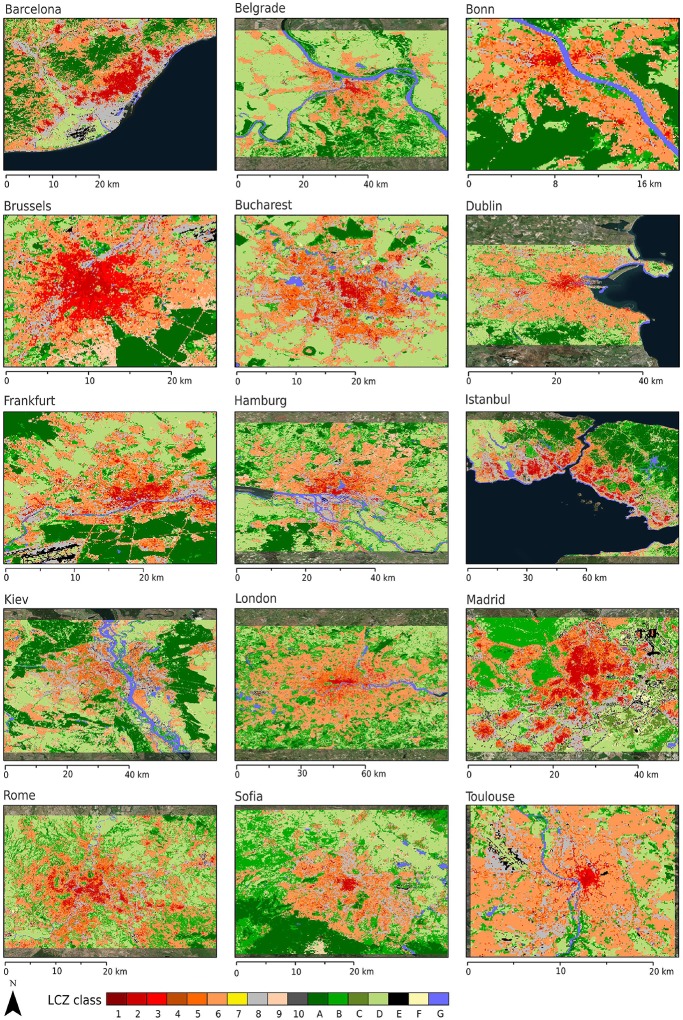
Local climate zones for 15 European cities. Sources background map: Esri, DigitalGlobe, Earthstar Geographics, CNES/Airbus DS, GeoEye, USDA FSA, USGS, Aerogrid, IGN, IGP, and the GIS User Community.

Fig 12 illustrates the SVF footprint for 15 European cities derived from GSV imagery, and can be viewed in relation to their corresponding LCZ maps shown in Fig 13. In general, reduced SVFs can be observed in the urban core except for Bonn (Germany), which has a relatively low-rise core owing to its historical development. Barcelona, Brussels and Hamburg exhibit the lowest mean SVF with 70%, while Toulouse and Dublin have the highest mean SVF at 84%. Table 6 summarises mean SVFs for each of the built LCZ types for each city. The comparison of average SVFs in each zone to LCZ values for all cities shows excellent results considering the spatial scope of the LCZ map. The values for Open low-rise (LCZ 6) and Large low-rise (LCZ 8) are well within the LCZ ranges for all cities but the values for compact types are generally higher from GSV: 23% higher for high-rise (LCZ 1, just once city) and 10% and 14% higher for mid- and low-rise, respectively. This could be linked to the underestimation of building height values by the WUDAPT methodology discussed above, which affects the LCZ classification and, as a consequence the associated SVF value. This is also seen in the confusion between some LCZ types, such as compact and open mid-rise (LCZ 2 and 5), which might indicate that some neighbourhoods are perceived as more compact than they are in the creation of TA data. However, some of the discrepancies may be a result of the GSV methodology, which has been shown to overestimate SVF in densely built parts of cities due to incomplete spatial sampling that does not include backyards and courtyards [63, 66].

**Table 6:** Mean sky view factor (%) per built LCZ class. Reference values from [18] are provided in the top row, mean and standard deviation (St. Dev.) across all selected cities are provided on top of the individual city listings.

	LCZ 1	LCZ 2	LCZ 3	LCZ 4	LCZ 5	LCZ 6	LCZ 7	LCZ 8	LCZ 9	LCZ 10
[18]	20-40	30-60	20-60	50-70	50-80	60-90	20-50	>70	>80	60-90
**Mean**	63.8	70.1	74.1	69.8	76.3	78.2	-	80.0	78.9	85.3
**St. Dev**.	0.0	3.7	5.6	9.5	4.3	4.1	-	3.4	6.8	5.7
Barcelona	-	65.2	65.0	53.7	71.5	77.0	-	75.9	74.9	77.3
Belgrade	-	71.7	74.7	70.0	75.4	79.8	-	82.6	84.3	-
Bonn	-	71.3	71.3	74.3	75.0	74.8	-	80.2	73.4	-
Brussels	63.8	63.8	69.4	66.6	70.8	71.0	-	77.0	67.8	-
Bucharest	-	72.4	83.5	64.4	82.9	83.6	-	82.9	84.7	-
Dublin	-	78.9	81.2	86.6	85.7	85.4	-	87.6	82.8	85.3
Frankfurt	-	67.7	68.1	74.4	74.7	78.5	-	77.4	80.2	-
Hamburg	-	67.0	73.5	76.9	70.6	71.8	-	76.9	69.5	79.0
Istanbul	-	68.5	68.8	69.0	77.0	81.4	-	76.3	89.4	83.3
Kiev	-	66.8	72.0	75.1	74.8	75.3	-	77.7	76.2	89.9
London	-	71.1	79.9	71.9	78.6	75.3	-	79.5	71.3	83.4
Madrid	-	72.9	77.1	60.5	74.1	79.6	-	82.3	88.0	96.7
Rome	-	69.0	77.6	50.1	74.8	76.4	-	77.4	75.2	-
Sofia	-	73.5	80.9	69.6	76.0	80.3	-	81.1	87.8	82.7
Toulouse	-	72.0	67.8	83.3	82.4	83.3	-	85.1	78.3	90.0

The spherical fractions do not directly correspond to the reference values for LCZs due to different viewpoints of the land cover fractions (within canyon vs. bird’s eye view), yet they offer valuable insight into the urban form and composition of parameters as measured from the streets in each zone. Here these results are presented in the supplementary materials providing average spherical fractions for buildings, trees, impervious and pervious surfaces, sky, and non-permanent objects in each city, calculated after [64]. In summary:

- The spherical building fraction is based on walls (rather than roofs) and highlights the vertical dimension of urban form. Barcelona and Istanbul have the highest fractions among all cities (S2 Table), which is indicative of taller buildings, more compact developments, and/or less trees compared to the other cities under investigation.
- The spherical impervious fraction is the least variable and the most stable value across all LCZs and cities (S3 Table), because GSV image locations are biased towards the centre of the road. Only few GSV images exist in parks and other open areas hence the very low mean pervious fractions across all LCZ types (S4 Table).
- Sparsely built areas generally have the highest tree fraction, followed by open areas (LCZs 4, 5, and 6) (S5 Table). Fewest trees can be found in compact zones (LCZs 1, 2, and 3) and in Large low-rise (LCZ 8) areas, which are usually commercial spaces.
- Sparsely built areas (LCZ 9) show the biggest variability of tree cover, which reflects differences in climate and vegetative cover. For example, Barcelona’s LCZ 9 has 41.1% spherical tree coverage in a Mediterranean climate, while Madrid exhibits more of a semi-arid climate and has only 16.3% tree coverage in LCZ 9.
- Finally, the moving objects fraction is low for all zones (S6 Table). This class includes non-permanent urban features such as cars, trucks, trash bins, and pedestrians and reflects the level of anthropogenic activity in the street canyon at the time of GSV image acquisition. The fraction is highest in compact LCZs, which are usually in the city center (6.6%-7.6%), and lowest in Sparsely built areas (1.9%).

Overall, these results are in line with the expected characteristics of LCZ types.

## Summary and conclusion

This research has taken a major step towards the creation of a global scale urban database suited for climate studies by utilising the computational power and resources of EE and the experience derived from creating city-by-city LCZ maps using the standard WUDAPT protocol. This experience has informed an urban transferability strategy that, coupled with EE, permits the creation of a continental scale product.

Here, a training area dataset was carefully compiled from existing WUDAPT data gathered by a number of individuals for a number of European cities and their surrounding natural environments. These data were used by a random forest classifier to create LCZ types from input features derived from geospatial datasets (mostly, multi-year series of multispectral and SAR satellite observations). All experiments reach overall accuracy scores above 70%, which fulfils the requirement of an average minimum accuracy of 50% to pass the automated quality control performed in WUDAPT [21]. The best performing experiment used all training information and all input features and resulted in overall accuracies of more than 80% for both urban and natural LCZ types. Moreover, a simple urban/non-urban mask showed a very high agreement with the most recent ESA CCI land cover map. Of the input features, those derived from Sentinel-1 SAR data and various spectral indices had the strongest discriminating power. This work has also provided guidance for other up-scaling experiments, notably that greater effort should be given to including more input features over acquiring more sample pixels per training area polygon.

In addition, country specific parameter sets for model applications for building height, impervious density, mean annual anthropogenic heat flux, and sky view factor were derived using the European LCZ map and auxiliary data, which for some properties are only available for selected cities. While the derived parameters mostly match the ranges provided by [18], some deviations need further investigation. In particular the derived building heights show considerable confusion between mid- and low-rise classes, but also some of the parameter ranges such as LCZ 8 building height and impervious surface fractions for the compact LCZ classes may need refinement.

This study proved that large scale LCZ mapping is possible in a cloud computing environment given sufficient training data for the respective region. Subsequent studies need to address the lack of training data for other regions of the world (such as Africa and India), that is the current impediment to creating a global map. This work has shown that it is possible to generate a consistent and complete continental-scale LCZ map, which can serve the needs of global climate science.

## Supporting information

S1 Table List of abbreviations. (PDF)

S2 Table Mean building fraction (%) per built LCZ class. (PNG)

S3 Table Mean impervious fraction (%) per built LCZ class. (PNG)

S4 Table Mean pervious fraction (%) per built LCZ class. (PNG)

S5 Table Mean tree fraction (%) per built LCZ class. (PNG)

S6 Table Mean ‘moving’ fraction (%) per built LCZ class. (PNG)
