# Supplementary material for: Mapping Europe into local climate zones
Source: PLoS One. 2019 Apr 24;14(4):e0214474. doi: 10.1371/journal.pone.0214474 (PMC6481911; doi:10.1371/journal.pone.0214474)
Supplement: S1 Table — (PDF) [file pone.0214474.s001.pdf]

S1 Table. List of abbreviations

Abbreviation	Explanation
AHF	Anthropogenic heat flux [W m^{-2}]
B2	Blue Landsat 8 band
B3	Green Landsat 8 band
B4	Red Landsat 8 band
B5	Infrared Landsat 8 band
B6/B7	Shortwave infrared Landsat 8 bands
B10/B11	Thermal infrared Landsat 8 bands
BCI	Biophysical Composition Index
BH	Building height
CLMS	Copernicus Land Monitoring Service
CMIP5/6	The fifth/sixth Climate Model Intercomparison Project
DEM/DSM/DTM	Digital elevation model/Digital surface model/ Digital terrain model
DMSP/OLS	Defense Meteorological Program/ Operational Linescan System
EBBI	Enhanced Built-up and Bare land Index
EE	Google Earth Engine
EEA	The European Environmental Agency
EROS	Earth Resources Observation
ESA CCI	European Space Agency Climate Change Initiative
GFCH	Global Forest Canopy Height
GHSL	Global Human Settlement Layer
GSV	Google Street View
GUF	Global Urban Footprint
H	Mean height of roughness elements (m)
HighResMIP	High Resolution Model Intercomparison Project
HUMINEX	Human influence experiment
IMD	Impervious surface density (%)
JRC	European Joint Research Center
JtD	January to December
L8	Landsat 8
LC	Local Cover
LCZ	Local Climate Zone
LSIB	Large Scale International Boundary
MtS	May to September
NDBAI	Normalized Difference BAreness Index
NDBI	Normalized Difference Built Index
NDUI	Normalized Difference Urban Index
NDVI	Normal Difference Vegetation Index
NDWI	Normalized Difference Water Index
NtM	November to March
OA	Overall accuracy
OA_{bu}	Overall accuracy of urban versus natural LCZ classes
OA_u	Overall accuracy for urban LCZ classes
OA_w	Weighted overall accuracy
OSM	OpenStreetMap
SAR	Synthetic aperture radar
SVF	Sky view factor (0-1)
TA	Training area
TPR	True Positive Rate
UCP	Urban Canopy Parameter
UN-LCCS	United Nations Land Cover Classification System
WUDAPT	World Urban Database Access Portal Tools
λ_B	Building fraction (%)
λ_I	Impervious ground fraction (%)
λ_V	Pervious ground fraction (%)
