# Supplementary figures and images for: Mapping Europe into local climate zones
Source: PLoS One. 2019 Apr 24;14(4):e0214474. doi: 10.1371/journal.pone.0214474 (PMC6481911; doi:10.1371/journal.pone.0214474)

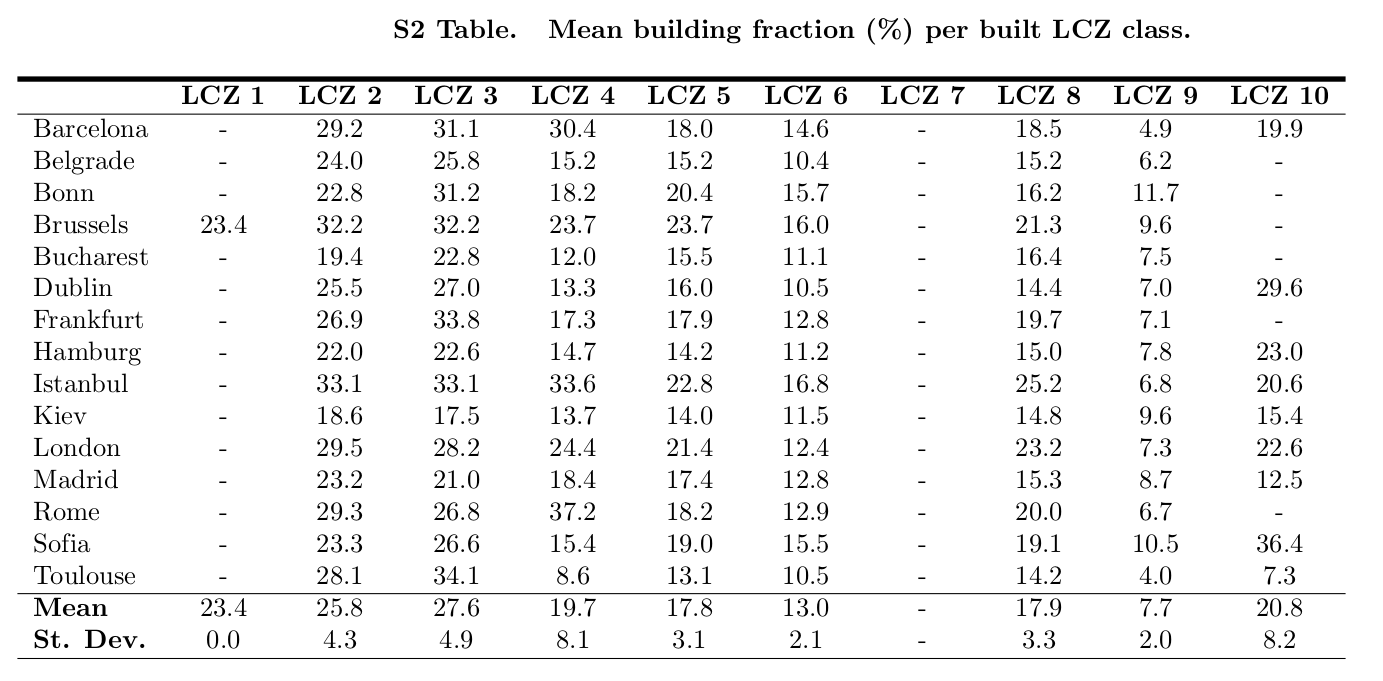

Supplement: S2 Table — (PNG) [file pone.0214474.s002.png]

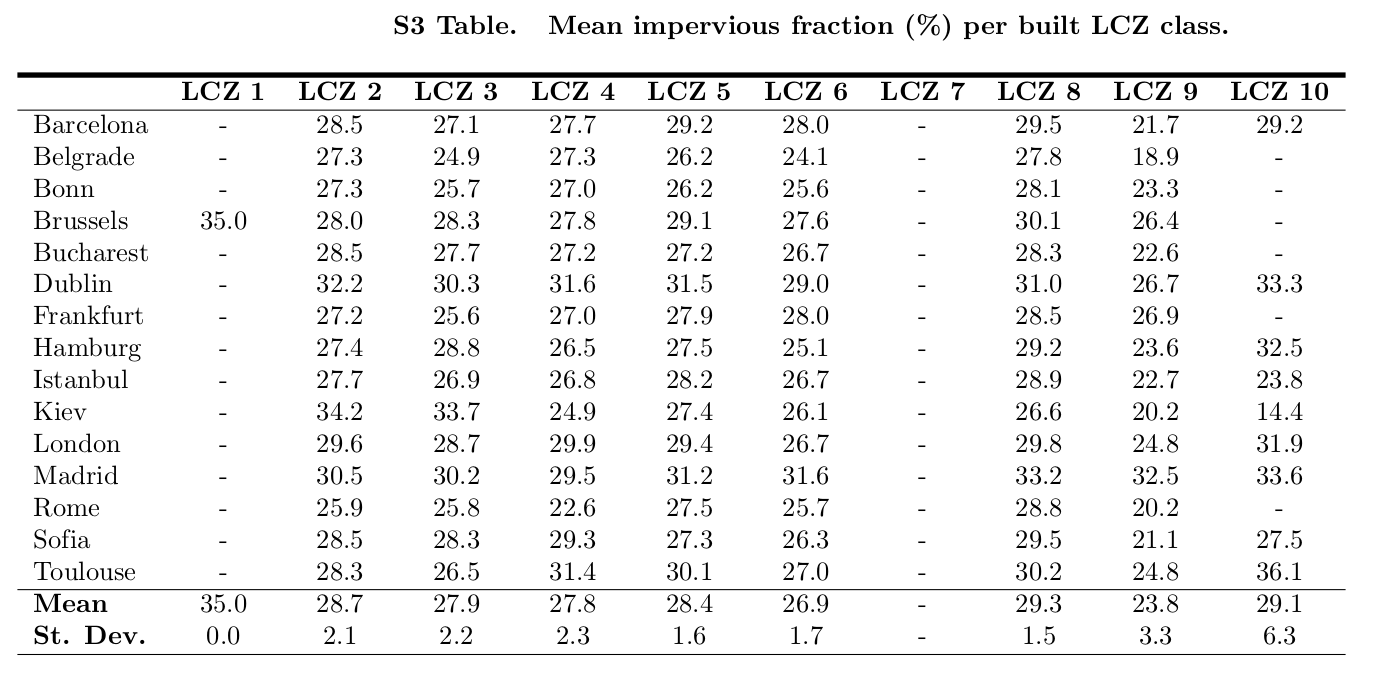

Supplement: S3 Table — (PNG) [file pone.0214474.s003.png]

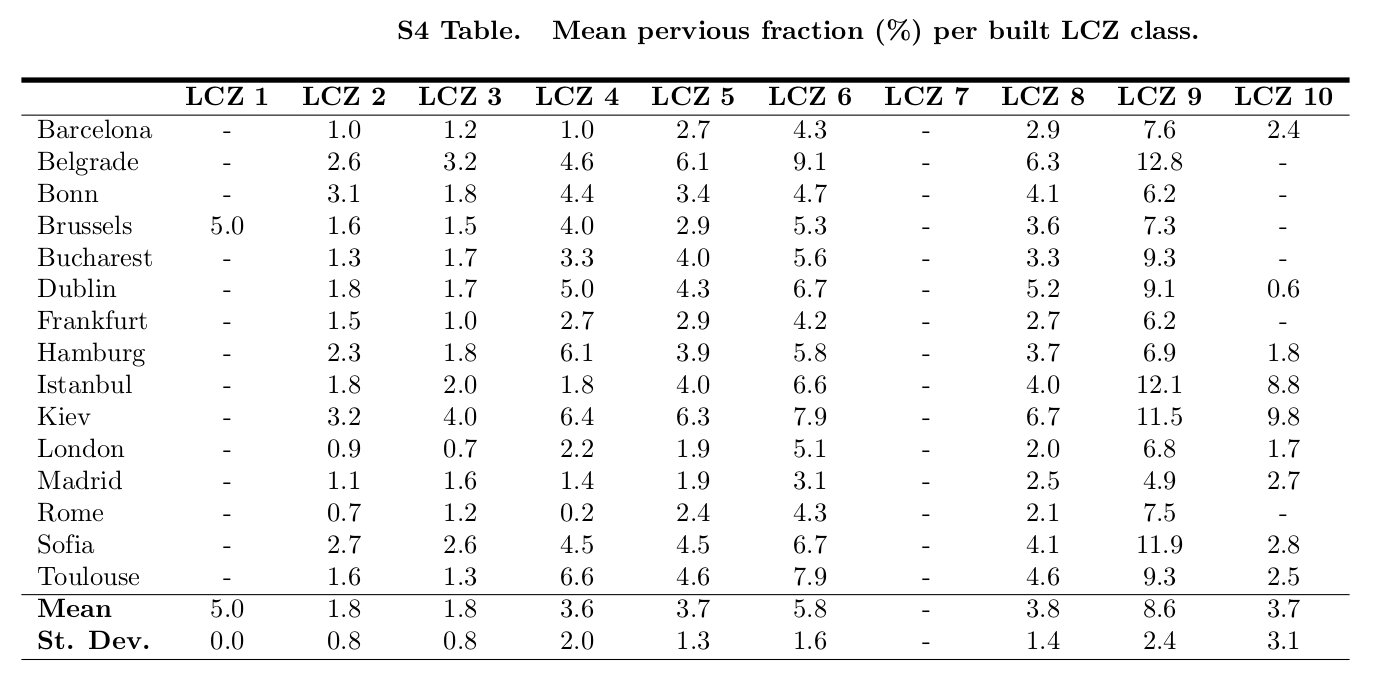

Supplement: S4 Table — (PNG) [file pone.0214474.s004.png]

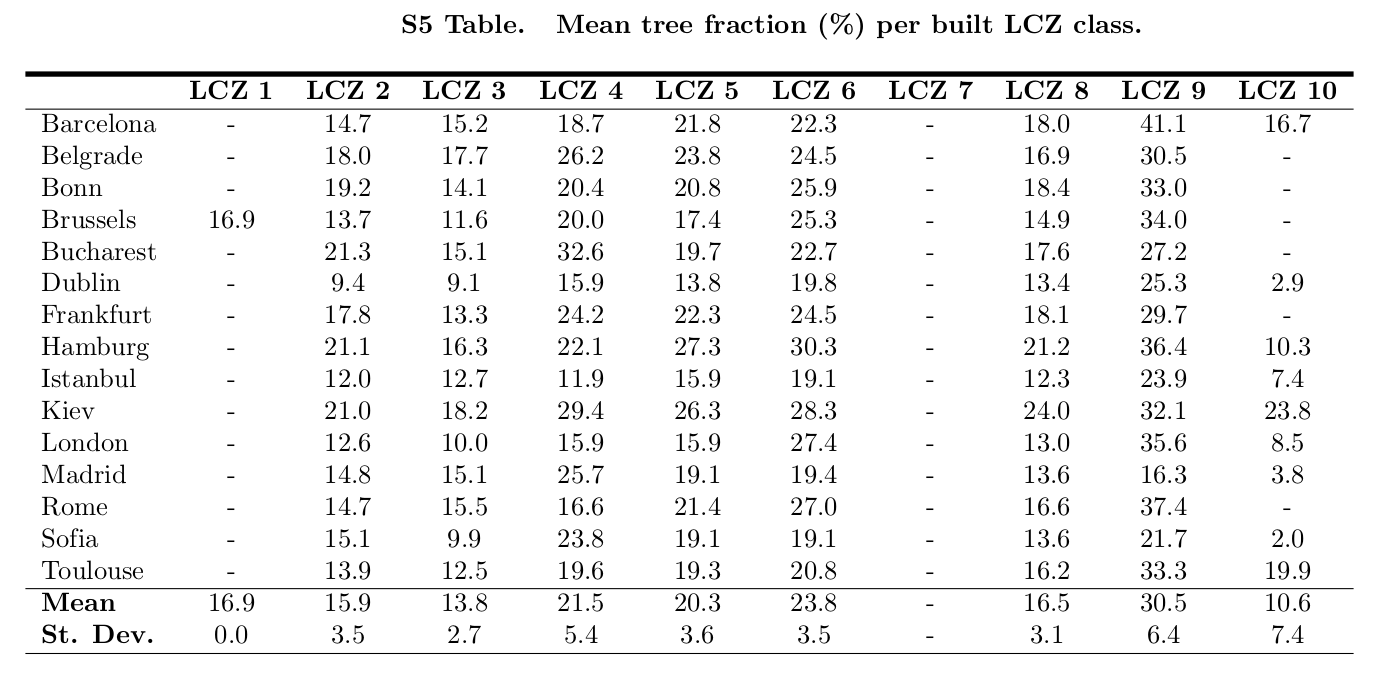

Supplement: S5 Table — (PNG) [file pone.0214474.s005.png]

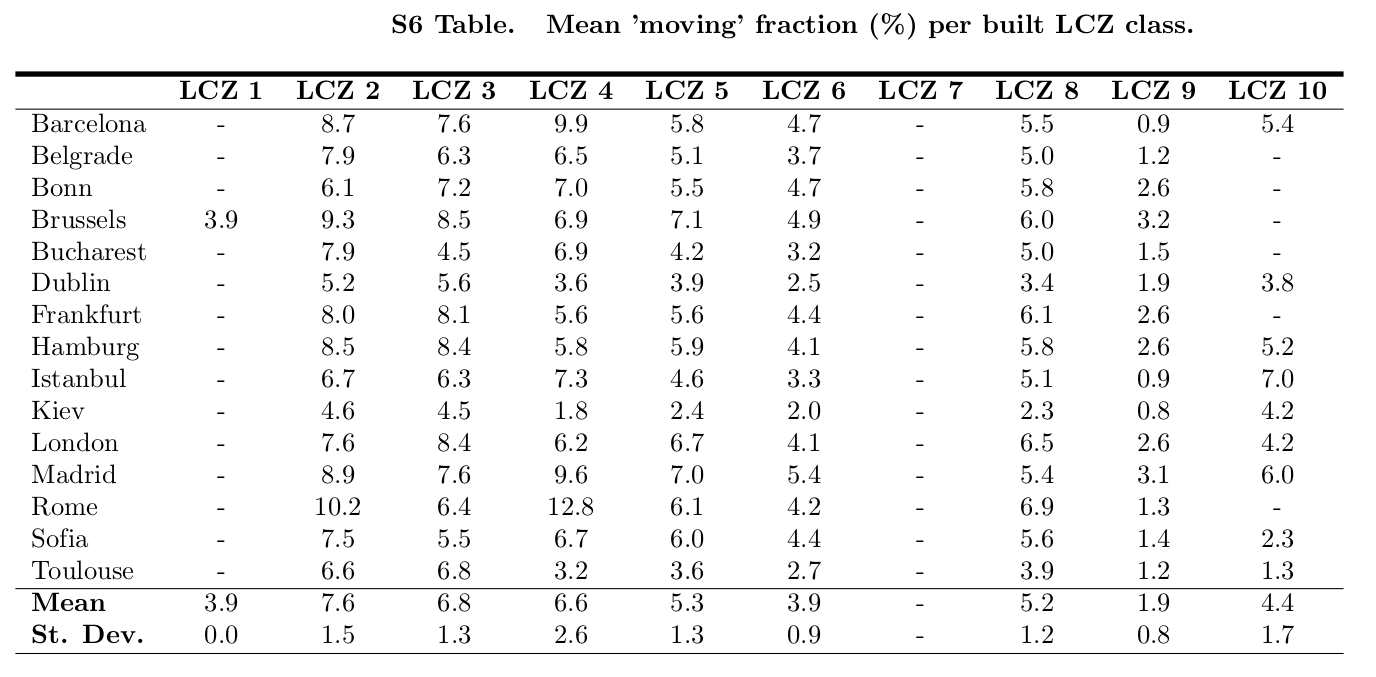

Supplement: S6 Table — (PNG) [file pone.0214474.s006.png]
